# Implementation of circulating tumour DNA multi-target mutation testing in plasma: a perspective from an external quality assessment providers’ survey

**DOI:** 10.1007/s00428-023-03558-x

**Published:** 2023-05-19

**Authors:** Jennifer A. Fairley, Tony Badrick, Marc G. Denis, Lora Dimitrova, Rebecca Goodall, Joerg Maas, Nicola Normanno, Simon J. Patton, Etienne Rouleau, Antonio Russo, Tracy L. Stockley, Zandra C. Deans

**Affiliations:** 1 GenQA, Department of Laboratory Medicine, NHS Lothian, Nine Bioquarter, Little France Rd, Edinburgh, EH16 4UX UK; 2 The Royal College of Pathologists of Australasia, Quality Assurance Programs (RCPAQAP), St. Leonards, Australia; 3 Nantes Université, CHU Nantes, Department of Biochemistry, INSERM, CNRS, Immunology and New Concepts in Immunotherapy, Nantes, France; 4 Quality in Pathology (QuIP GmbH), Berlin, Germany; 5 EMQN CIC, Unit 4, Enterprise House, Manchester Science Park, Pencroft Way, Manchester, M15 6SE UK; 6 Deutsche Gesellschaft für Pathologie E.V. (DGP), Berlin, Germany; 7 Cell Biology and Biotherapy Unit, Istituto Nazionale Tumori - IRCCS - “Fondazione G. Pascale”, Via Mariano Semola, 80131 Napoli, Italy; 8 Department of Medical Biology and Pathology, Gustave Roussy, Cancer Genetics Laboratory, Gustave Roussy, 94800 Villejuif, France; 9 Department of Surgical, Oncological and Oral Sciences, Section of Medical Oncology, University of Palermo, 90127 Palermo, Italy; 10 Laboratory Medicine Program, University Health Network; Advanced Molecular Diagnostics Laboratory, Princess Margaret Cancer Centre, Department of Laboratory Medicine and Pathobiology, University of Toronto, Toronto, Ontario Canada

**Keywords:** Large-scale tumour profiling, Circulating tumour DNA, EQA scheme

## Abstract

Demand for large-scale tumour profiling across cancer types has increased in recent years, driven by the emergence of targeted drug therapies. Analysing alternations in plasma circulating tumour DNA (ctDNA) for cancer detection can improve survival; ctDNA testing is recommended when tumour tissue is unavailable. An online survey of molecular pathology testing was circulated by six external quality assessment members of IQN Path to registered laboratories and all IQN Path collaborative corporate members. Data from 275 laboratories across 45 countries were collected; 245 (89%) perform molecular pathology testing, including 177 (64%) which perform plasma ctDNA diagnostic service testing. The most common tests were next-generation sequencing-based (*n* = 113). Genes with known stratified treatment options, including *KRAS* (*n* = 97), *NRAS* (*n* = 84)*,* and *EGFR* (*n* = 130), were common targets. The uptake of ctDNA plasma testing and plans to implement further testing demonstrates the importance of support from a well-designed EQA scheme.

**Supplementary Information:**

The online version contains supplementary material available at 10.1007/s00428-023-03558-x.

## Introduction

Advanced cancer patients have poor survival. Therefore, there is a need for more widespread implementation of simple, accurate, and non-invasive techniques for the detection of cancers [1, 2] and the rapid detection of molecular biomarkers to drive the selection of targeted treatments [3] possible using circulating tumour DNA (ctDNA) testing. Detection of molecular alterations in ctDNA has many potential clinical applications [4] including cancer screening, treatment monitoring, detection of minimal residual disease, and molecular relapse monitoring [5]. Liquid biopsies can detect multiple cancers and, in some cases, identify the tissue of origin whilst offering genomic testing through a minimally invasive process. The analysis of genomic and epigenetic alterations in ctDNA as a fraction of plasma circulating cell-free DNA has been demonstrated to facilitate subsequent diagnosis and improve survival [2, 6]. Testing of ctDNA is recommended when tumour tissue is not available for genomic profiling, or when rapid results are clinically important [5].

In 2017, we conducted a survey to assess the standard of ctDNA testing [7]. Our previous study evaluated laboratory practices, which informed the design of a pilot external quality assessment (EQA) for plasma ctDNA testing. Since 2017, there has been a rapid expansion in the number of predictive molecular biomarkers and associated targeting therapies. This has increased the need for prospective tumour profiling across all cancer types.

Our 2017 survey reported that the most frequently used method of plasma ctDNA testing was next-generation sequencing (NGS), used by 27% of surveyed laboratories [7] which, alongside the rising number of large-scale tumour molecular profiling programs worldwide, has revolutionised the field of precision oncology [8].

In 2017, many laboratories planned to implement ctDNA testing [7]. In light of this, we conducted a follow-up survey in 2021 incorporating questions regarding ctDNA multi-target mutation testing in blood plasma. The aim was to review the liquid biopsy testing landscape regarding methodologies and gene targets that were likely to have been implemented by the same laboratories during the previous 5–6 years. This report summarises the survey results, which evaluated the current practice of gene testing in liquid biopsies.

## Methods

The design of the survey was informed by our earlier online survey that reviewed ctDNA testing practice, designed by GenQA and reviewed by the IQN Path collaborative group [7].

The current survey was circulated by EQA member organisations of IQN Path to their registered laboratories: EMQN, AIOM, GenQA, RCPAQAP, QuIP, CBQA, Gen&Tiss/GFCO, and to all corporate members of the IQN Path collaborative (pharmaceutical and diagnostic companies). The survey comprised 32 questions that collected data on laboratory testing (molecular pathology testing/diagnostic clinical service using ctDNA testing), methodologies for ctDNA testing, the genes and specific variations being tested and plans for implementing testing, and experiences associated with testing (test quantities, timelines, plasma types).

The survey opened for completion between July 1, 2021 and August 20, 2021. The responses were analysed to understand the implemented practices of single-gene testing in liquid biopsies (availability, methodology, data accuracy, and variability).

## Results

### Survey population

**Fig. 1:**
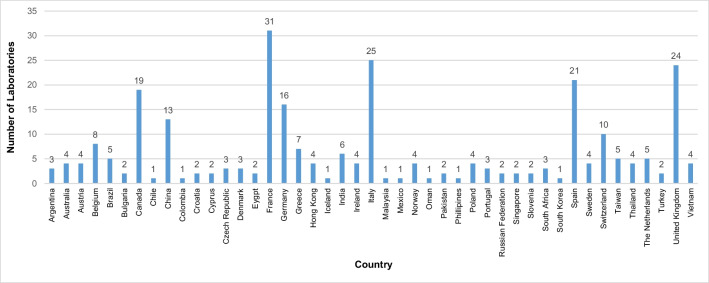
Number of laboratories per country from respondents who provided their location (*n* = 272)*. Values represent the number of participating laboratories per country. *A total of 3 laboratories out of the total 275 laboratories did not provide a response regarding the country they are located

Completed surveys were received from 275 laboratories in 45 different countries. The highest number of returned surveys were from France, Italy, the United Kingdom, Spain, and Canada (Fig. 1). Survey responses were collated and analysed descriptively.

A total of 161 out of the 275 laboratories surveyed reported their type of company. The majority of respondees (*n* = 85) were clinical testing laboratories, followed by diagnostic manufacturers (*n* = 20) and pharmaceutical companies (*n* = 2). A total of 54 laboratories reported “other”, which included academic centres/universities, public hospitals, and research foundations.

### Molecular pathology testing

Out of 275 laboratories that shared data on this topic, 245 (89%) perform molecular pathology testing, whilst 29 (11%) do not — one laboratory did not provide information on this.

### Molecular pathology testing targets

A summary of responses on testing targets received by the participating laboratories is displayed in Supplementary Figure 1a. Responses were received from 273 laboratories, of which 149 laboratories detailed the targets tested. The survey data showed that in laboratories only testing one target, the most common approach was mutation hotspot testing (22 laboratories). In laboratories testing multiple targets, the most common combination of testing was single gene, multiple gene, full coding regions, and targeted mutation regions (22 laboratories).

Overall, targeted mutation hotspots were the most common targets for laboratories (*n* = 102), with the least common being full coding regions of genes (*n* = 37) (Supplementary Figure 1b) and 84 and 89 laboratories, respectively, tested single- and multiple-gene targets.

### ctDNA testing

There were 114 (41%) laboratories that reported the number of ctDNA diagnostic tests carried out in 2020 and their turn-around times. The majority of laboratories reportedly carried out <50 (*n* = 47, 41%) or 51–200 (*n* = 48, 42%) tests, with 12 reporting 201–500 tests, three reporting 501–1000, and four reporting >1000 tests. Three (3%) laboratories reported a turn-around time of either <1 day (<24 h) and four (4%) reported 2 days, with the majority reporting a time of either 7 days (*n* = 22, 22%) or 8–10 days (*n* = 24, 24%).

A total of 177 (64%) laboratories reported they offer a clinical diagnostic service using ctDNA testing, whilst 65 (24%) do not; 33 laboratories did not respond. Of those laboratories providing a diagnostic service (*n* = 177), most laboratories test in-house (*n* = 123), whilst 27 laboratories outsource their current clinical services, and 125 did not answer this question.

### Current and planned testing

Responses regarding current ctDNA testing were received by 214 laboratories, with 198 responding to future testing plans. At the time of the survey, 130 laboratories reportedly performed research using ctDNA testing, with 84 stating they did not and 61 laboratories did not respond. A total of 21 laboratories that perform research using ctDNA testing reported no plans to implement further testing in the future. Of the laboratories that did not currently perform ctDNA testing (*n* = 84), 60 indicated that they plan to implement ctDNA testing in the future.

### ctDNA testing methodologies

A total of 207 laboratories reported the testing methods used for ctDNA testing (Supplementary Figure 2). Data were not available for 56 laboratories. The most common testing method used by individual laboratories was NGS alone (*n* = 33), with 17 labs using amplicon-based NGS, 13 capture-based NGS, and three using both approaches. Real-time *polymerase chain reaction* (RT-*PCR*) was used independently of other methods by 25 laboratories. Of the 209 laboratories that reported testing methods, 74 reported using multiple methods, whilst 68 reported only using a single testing method.

Overall, RT-PCR was reported by 70 laboratories of which one laboratory each specified cobas^®^ and allele-specific PCR (Supplementary Figure 2b). This was followed by ddPCR (n = 64), BEAMing (n = 6), MassARRAY by three, and End-point PCR by one laboratory. NGS was reported by 112 laboratories; 71 laboratories reported the use of amplicon-based NGS, and 41 reported capture-based NGS (Supplementary Figure 2b).

### ctDNA target genes

#### EGFR gene targets

**Fig. 2:**
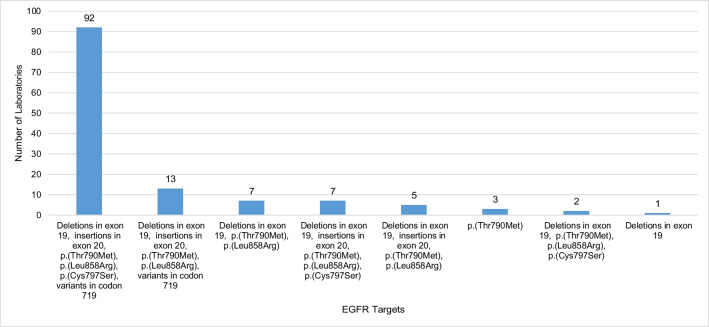
A breakdown of the *EGFR* gene targets tested within the diagnostic clinical service of laboratories*. *The number of laboratories who did not provide *EGFR* targets are not included in this figure (*n* = 72), in addition to those laboratories who do not perform circulating tumour DNA testing (*n* = 65). *EGFR* nomenclature according to NM_005228.5

Figure 2 illustrates *EGFR* (NM_005228.5) gene variants tested within the clinical diagnostic service of laboratories. A total of 130 laboratories tested the *EGFR* gene, whereas 72 did not include *EGFR* within the scope of their testing. The most common combination of gene testing targets was deletions in exon 19; insertions in exon 20, p.(Thr790Met), p.(Leu858Arg), and p.(Cys797Ser); and variants in codon 719, of which 92 laboratories applied this testing combination. The testing combination of deletions within exon 19; insertions in exon 20, p.(Thr790Met), and p.(Leu858Arg); and variants in codon 719 was applied by 13 laboratories. Only deletions within exon 19 were targeted by one laboratory and only p.(Thr790Met) was targeted by three laboratories; no other *EGFR* gene targets were reported to be targeted individually.

#### KRAS and NRAS gene testing

**Fig. 3:**
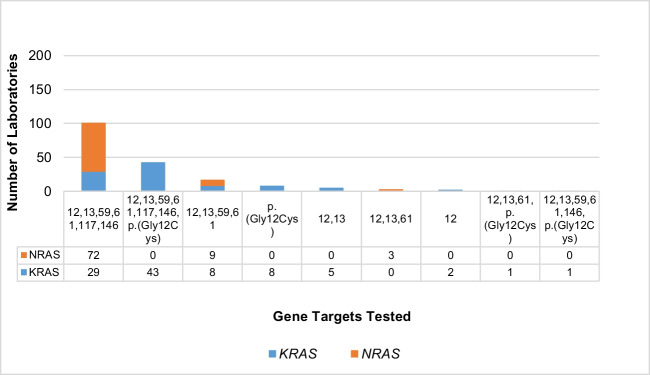
*KRAS* and *NRAS* gene targets tested within the diagnostic clinical service of laboratories. Nomenclature according to NM_004985.5 (*KRAS*) and NM_002524.3 (*NRAS*)

*KRAS* and *NRAS* variants tested by laboratories for both are presented in Fig. 3. A total of 272 laboratories reported whether they carry out gene testing; 97 laboratories reported testing for specific variants for *KRAS*, and 84 for *NRAS*.

The most common targets for *NRAS* (NM_002524.3) were codons 12, 13, 59, 61, 117, and 146 (72 laboratories), whereas the most commonly tested regions for *KRAS* (NM_004985.5) were codons 12, 13, 59, 61, 117, 146, and p.(Gly12Cys) specifically (43 laboratories). Codons 12, 13, 59, and 61 were identified as testing targets in both *KRAS* (*n* = 8) and *NRAS* (*n* = 9) genes.

#### Other gene targets

A total of 24 other targets were reportedly included in testing strategies amongst the surveyed laboratories. These gene targets are displayed in Supplementary Table 1. The most commonly tested target was *PIK3CA* (NM_006218.3)*,* with testing regions codon 542 (*n* = 88), codon 545 (*n* = 94), and codon 1047 (*n* = 93). Testing of *BRAF* (NM_004333.6) was also common across laboratories on exon 11 (*n* = 75), exon 15 (*n* = 86), and p.(Val600Glu) only (*n* = 59).

## Discussion

The survey data of 275 clinical laboratories worldwide indicates an increase in ctDNA testing in between 2017 and 2021. Our previous study [7] reported that only 37% of surveyed laboratories performed diagnostic plasma ctDNA testing in 2017, compared with 64% of laboratories testing ctDNA in 2021. The survey data also show an increased interest of laboratories to implement further ctDNA testing, with 56% of laboratories sharing plans to implement ctDNA testing, compared with 34% of laboratories in 2017 [7]. Our findings are reflected in the literature, which reports an increased number of investigations into to the utility of monitoring tumour genomics through plasma ctDNA analysis in a variety of clinical settings in recent years [9, 10]. ctDNA characterisation can aid in uncovering tumour-specific determinants; for example tumour mutational burden and the inclusion of standardised ctDNA assessments is recommended across cancer entities for personalised cancer immunotherapy to illustrate the clinical benefit of ctDNA as a biomarker for interventional clinical trials [11]. Furthermore, the uptake of ctDNA testing may be due to the ability to identify tumour specific abnormalities. Additionally, specific pathogenic variants in genes have been identified in the plasma of patients with several types of cancer, highlighting ctDNA as a potential cancer biomarker [12].

NGS was the most popular method for plasma ctDNA testing in 2017 [7]. This report found that NGS (54%) continues to be used more widely than RT-PCR (33%), likely due to the ability of NGS to simultaneously detect multiple mutations in various genes in a single test [13]. Amplicon-based NGS and capture-based library preparation for NGS were popular methods reported by laboratories independent of other testing methods. As predicted by previous reports [14] and shown here, RT-PCR is also a popular option for the analysis of cancer markers which may be due to the rapidity of results that this method offers. Furthermore, NGS technology is expensive, requires bioinformatic expertise, and may not be available in all laboratories.

Despite NGS and RT-PCR being commonly used, no specific testing method has emerged as the sole method preferred by laboratories; our previous conclusion in 2017 that no single, definitive technology for the analysis of plasma ctDNA has yet emerged [7] is still applicable today. Research suggests that further investigation is needed to increase the specificity and sensitivity of testing; however, the optimal sensivity for ctDNA testing is still not evident, as reflected in our 2017 study [7]. Therefore, improvements in ctDNA sensitivity are needed [15]. One study supports the use of targeted NGS in the screening of *EGFR*, *KRAS*, and *BRAF* mutations in formalin-fixed, paraffin-embedded tumour tissue compared to RT-PCR, as NGS revealed seven non-synonymous single-nucleotide variations and one insertion-deletion variation in *EGFR* which was not detectable by the RT-PCR methods [13]. Furthermore, some NGS approaches provide more accurate information on allele sequence, mutating frequency and detecting non-hotspot mutations when compared to PCR, depending on the panel used and the targeted RT-PCR assay, which may explain why NGS was the most popular method reported in our survey [16]. However, laboratory expertise, scale of economy, and the availability of a high-throughput NGS which can be utilised for other tests may also contribute to this observation. As expected, the most commonly tested genes are those with known stratified treatment options, i.e. *NRAS, KRAS*, and *EGFR.* However, this report identified various additional targets in routine use.

The relatively low number of laboratories providing ctDNA tests for all the approved biomarkers might lead to under-genotyping of a significant fraction of cancer patients, with particular regard to patients with lung adenocarcinoma for whom the use of NGS in clinical practice is strongly recommended [5, 17]. These findings also confirm the results of a recent survey showing significant limitations in the access to multigene biomarker testing in the majority of Europe which identified that <10% of specimens which require molecular testing are analysed with NGS [18]. As a result, access to precision medicines may be restricted due to limited biomarker testing access [18].

The uptake of ctDNA plasma testing methods and the increased interest of laboratories to implement further testing demonstrates the importance of support from a well-designed EQA scheme, of which there are several benefits. Participating laboratories are provided with the opportunity to review the comparison of both performance and results across different testing sites, objective evidence of testing quality, offers potential warning for issues associated with testing kits, and also provides indications of areas in need of improvement. Furthermore, participating in an EQA aids in assuring valued customers (for example clinical teams, patients, health authorities, and commissioners) that results are reliable [19] and continued participation prevents any concerns regarding test quality [18].

## Supplementary information

ESM 1 (DOCX 59 kb)
