# Supplementary material for: Implementation of circulating tumour DNA multi-target mutation testing in plasma: a perspective from an external quality assessment providers’ survey
Source: Virchows Arch. 2023 May 19;485(4):717–22. doi: 10.1007/s00428-023-03558-x (PMC11522039; doi:10.1007/s00428-023-03558-x)
Supplement: Supplementary file 1 — (DOCX 59 kb) [file 428_2023_3558_MOESM1_ESM.docx]

# Supplementary Materials

**Supplementary** **figure 1 a** The breakdown of testing targets combinations reportedly used in individual laboratories for molecular pathology testing. Values represent the number of laboratories using a specific testing target combination. **b.** The breakdown of testing targets reportedly used across laboratories for molecular pathology testing. Values represent the number of laboratories using a specific testing target.

**a**

**b**

**Supplementary figure 2 a** A breakdown of methodologies reportedly used for ctDNA mutation testing in plasma samples. Values represent the number of laboratories carrying out a specific combination of testing methodologies. Some laboratories use more than one testing method. **b.** A breakdown of methodologies reportedly used for ctDNA mutation testing in plasma samples. Values represent the number of laboratories carrying out a specific testing methodologies. Some laboratories use more than one testing method.

**a**

**b**

CtDNA, circulating tumour DNA; BEAMing, beads, emulsion, amplification and magnetics; ddPCR, droplet digital polymerase chain reaction; NGS, next generation sequencing; PCR, polymerase chain reaction

CtDNA, circulating tumour DNA; BEAMing; beads, emulsion, amplification and magnetics; NGS, next generation sequencing; PCR, polymerase chain reaction

**Supplementary table 1** A breakdown of additional gene targets tested within the diagnostic clinical service of laboratories

| Gene Target | Number of Laboratories |
| --- | --- |
| *PIK3CA* codon 545 | 94 |
| *PIK3CA* codon 1047 | 93 |
| *PIK3CA* codon 542 | 88 |
| *BRAF* exon 15 | 86 |
| *BRAF* exon 11 | 75 |
| *ERBB2* exon 20 | 72 |
| *MET* exon 14 skipping | 59 |
| *BRAF* p.(Val600Glu) | 59 |
| *KIT* exon 17 | 54 |
| *KIT* exon 11 | 53 |
| *KIT* exon 13 | 53 |
| *ALK* gene fusions | 51 |
| *IDH1* gene | 48 |
| *ROS1* gene fusions | 46 |
| *FGFR3* gene | 46 |
| *MET* amplification | 45 |
| *RET* rearrangement | 43 |
| *NTRK* gene fusions | 31 |
| *BRCA1* single nucleotide variants | 30 |
| *BRCA2* single nucleotide variants | 30 |
| *ATM* gene | 22 |
| *BRCA1* copy number variants | 21 |
| *BRCA2* copy number variants | 20 |
| *CDK12* gene | 20 |
